# Reversible Decrease of Portal Venous Flow in Cirrhotic Patients: A Positive Side Effect of Sorafenib

**DOI:** 10.1371/journal.pone.0016978

**Published:** 2011-02-14

**Authors:** Romain Coriat, Hervé Gouya, Olivier Mir, Stanislas Ropert, Olivier Vignaux, Stanislas Chaussade, Philippe Sogni, Stanislas Pol, Benoit Blanchet, Paul Legmann, François Goldwasser

**Affiliations:** 1 Center for Research on Angiogenesis Inhibitors (CERIA), Hôpital Cochin, AP-HP, Paris, France; 2 Department of Medical Oncology, Teaching Hospital Cochin, Paris, France; 3 Université Paris Descartes, Paris, France; 4 Department of Radiology, Teaching Hospital Cochin, Paris, France; 5 Gastroenterology Unit, Teaching Hospital Cochin, Paris, France; 6 Liver Unit and Inserm U-567, Hôpital Cochin, AP-HP, Paris, France; 7 Laboratory of Pharmacology, Hôpital Cochin, AP-HP, Paris, France; National Cancer Institute, United States of America

## Abstract

Portal hypertension, the most important complication with cirrhosis of the liver, is a serious disease. Sorafenib, a tyrosine kinase inhibitor is validated in advanced hepatocellular carcinoma. Because angiogenesis is a pathological hallmark of portal hypertension, the goal of our study was to determine the effect of sorafenib on portal venous flow and portosystemic collateral circulation in patients receiving sorafenib therapy for advanced hepatocellular carcinoma. Porto-collateral circulations were evaluated using a magnetic resonance technique prior sorafenib therapy, and at day 30. All patients under sorafenib therapy had a decrease in portal venous flow of at least 36%. In contrast, no specific change was observed in the azygos vein or the abdominal aorta. No portal venous flow modification was observed in the control group. Sorafenib is the first anti-angiogenic therapy to demonstrate a beneficial and reversible decrease of portal venous flow among cirrhotic patients.

## Introduction

Cirrhosis is a major public health problem, most commonly caused by alcoholism or viral hepatitis. Complications from advanced cirrhosis include hepatocellular carcinoma (HCC) and portal hypertension. Portal hypertension is characterized by a increased blood flow in the splanchnic organs draining into the portal vein and by the formation of porto-systemic collateral vessels, including gastroesophageal varices that can rupture and cause life-threatening bleeding[1], [2] Current pharmacotherapy for portal hypertension is limited to beta-blockers, but these drugs have an unpredictable response and can cause significant adverse events. [1], [3], [4]

One of the underlying causes of cirrhotic portal hypertension is the growth of collateral circulation [2]. In recent years, it has become increasingly evident that disturbances in the liver microcirculation, hypoxia and angiogenesis may occur in the injured liver and that angiogenesis plays a key role in the progression of liver fibrosis [5]. In experimental models of portal hypertension, a number of receptor tyrosine kinase inhibitors, including imatinib, sunitinib and sorafenib, have been shown to regulate splanchnic neovascularization and improve portal hypertension [6], [7]. Hence, receptor tyrosine kinase inhibitors offer a promising new approach to the management of portal hypertension.[8], [9]

Sorafenib (BAY-43-9006 Nexavar®, Bayer Pharmaceuticals Corp., Wayne NJ and Onyx Pharmaceuticals Inc., Emeryville CA), an oral multikinase inhibitor of the VEGF and the PDGF receptors and Raf, decreases tumor growth and inhibits angiogenesis in advanced HCC [10], [11], [12]. Sorafenib is already in clinical use as an anticancer drug that targets tumour cell proliferation and angiogenesis [11] and is approved for treatment of renal cell carcinoma [13] and for HCC [10]. Also, sorafenib has demonstrated clinical activity in various malignancies, including lung cancer, [14] thyroid cancer, [15] and soft tissue sarcomas [16], [17]. In advanced HCC (Child–Pugh class A), sorafenib is the only nonsurgical and nonradiological treatment to have demonstrated efficacy in improving survival in this disease.

However, sorafenib can lead to endothelial injury and promote vascular leakage, and is not approved for patients with portal hypertension complicated by cirrhosis of the liver and advanced HCC (Child–Pugh class B to C), even in the absence of gastrointestinal bleeding. [10], [11], [12]

It has been recently demonstrated in preclinical studies that sorafenib had a beneficial effect on porto-collateral circulation in cirrhotic animal with portal hypertension. [8], [18] However, no data have been presented at this time in humans. We report here portocollateral circulation changes in cirrhotic patients with advanced HCC treated with sorafenib.

## Methods

### Patient Population

Seven patients with advanced-stage HCC and portal hypertension were treated with sorafenib, at a validated dose of 400 mg twice daily until there was evidence of disease progression. Sorafenib was administered at 50% of the planned dose if any severe adverse events related to the study drug occurred, and in frail patients.[10] In sorafenib group, treatment interruptions and up to two dose reductions (first to 400 mg once daily and then to 400 mg every 2 days) were done in case of drug-related adverse effects. If further dose reductions were required, patients were withdrawn from the study. Treatment continued until the occurrence of either radiologic progression, as defined by RECIST criteria [19] or symptomatic progression.

Patients were included in the study if they fulfilled inclusion criteria and agreed to undergo repeat Magnetic Resonance Imaging during follow-up. None of the patients included in the present study received beta-blockers, in order to avoid confusion in the respective roles of beta-blockers and sorafenib on portal venous flow. Seven patients received at least one month of sorafenib therapy and underwent a second Magnetic Resonance Imaging. In a control group, the first nine patients who fulfilled the inclusion criteria and accepted repeated Magnetic Resonance Imaging were included.

An evaluation of porto-collateral circulations using a magnetic resonance technique was done before starting treatment, and at day 30. Five out of seven had a post therapy evaluation 30 days after withdrawal of sorafenib. To evaluate porto-collateral modification in cirrhotic patients, nine cirrhotic patients (control group) received an evaluation of porto-collateral circulations using a magnetic resonance technique was done at day 0 and day 30. All patients presented a normal blood pressure and no patients received beta-blocker therapy.

### Assessment of Blood Flow

**Figure 1:**
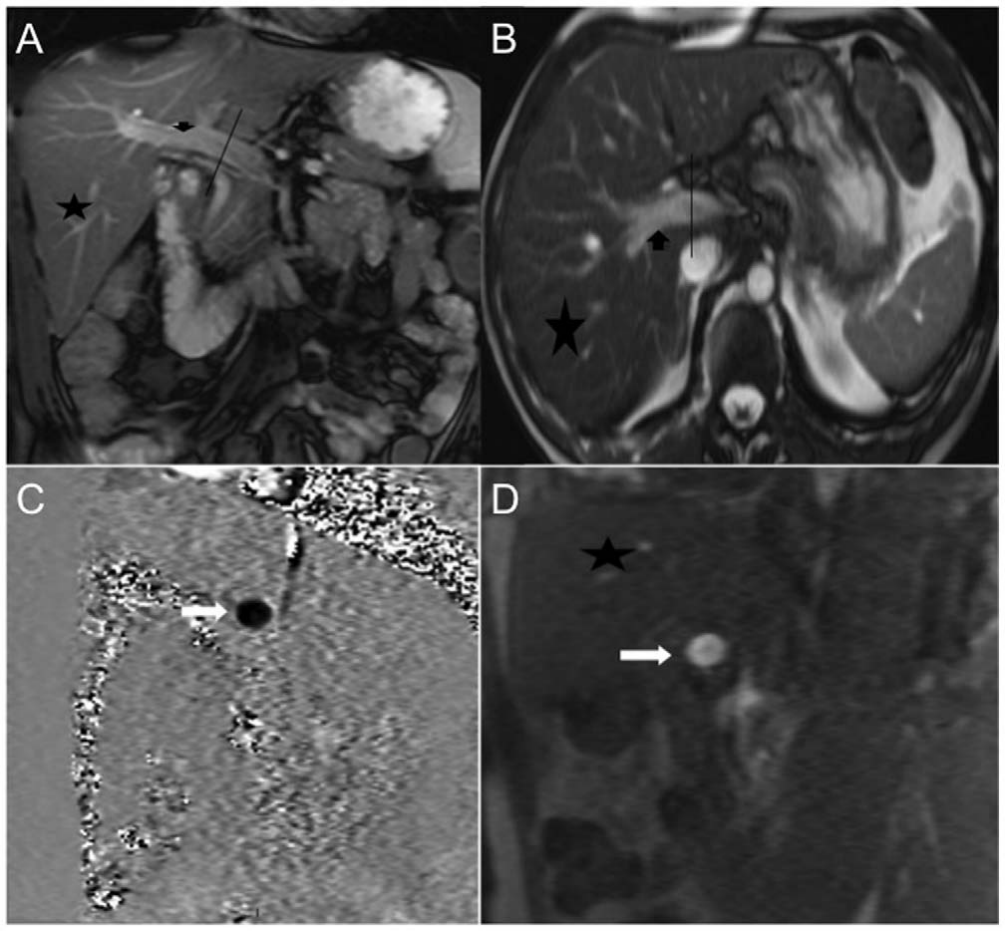
Female Patient, Aged 56 Years (Patient 2). Coronal (a) and transaxial (b) localizing image planned along the course of the portal vein (line). Based on these images, a phase-contrast enconding sequence was planned perpendicular to the course of the portal vein (line). c: Portal vein (arrowhead) velocity-encoded phase-contrast image. d: Portal vein (arrow) magnitude image.

Flow in the azygos vein and the portal venous systems were quantified with cine-phase contrast magnetic resonance imaging velocity mapping. [20], [21] Magnetic resonance imaging examinations were performed with a 1.5 T MR unit (Siemens Medical Solutions, Avanto, Erlangen, Germany) with electrocardiographic gating, using a cardiac dedicated 32-channel phased-array coil, and parallel acquisition to reduce the duration of acquisition. The time resolution was 16 frames in one cardiac cycle. Azygos flow was measured at the mid-thoracic level. Anatomical evaluation of the azygos and portal venous systems with axial, coronal, and oblique breath-hold sequences was performed to ensure the correct acquisition plane, perpendicular to the vein for flow quantification. Each set was reconstructed to yield a magnitude image and a velocity encoded phase-contrast image. Portal flow was measured in the main segment 20 mm proximal to the portal bifurcation. Volumetric flow rate was obtained from the product of the cross-sectional area and the velocity. Hand-drawn circular regions of interest were placed on the magnitude images so that all pixels of the vessel were included (Fig. 1).

Magnetic Resonance Imaging using phase-contrast velocity allows for direct non-invasive quantification evaluation of flow dynamics. Magnetic resonance Imaging was used to prevent flow variability measurements and to allow azygos venous flow evaluation. Phase-contrast Magnetic Resonance Imaging flow has the advantage to avoid intravenous injection of contrast media in this frail population. Phase-contrast flows measurements have been validated in vitro [22] as well as in vivo, including as an evaluation technique of the portal [23] and azygos venous flows [24]. Also, Magnetic Resonance Imaging Phase-contrast is a reproducible technique compared to ultrasonographic examination, which is a simpler technique but suffered from a high variability in repeated measurements. The Magnetic Resonance Imaging technique could help to improve Doppler flow calculations, thereby allowing standardization of protocols.

## Results

### Patient Characteristics

Between October 2009 and July 2010, 7 patients with advanced-stage HCC received sorafenib therapy according to the schedule described above and 9 patients were included in the control group. The patients' characteristics are shown in Table 1. Both in sorafenib group and in control group, the disease at baseline was rate as Child-Pugh class A or B. Chronic hepatitis C virus and alcohol were the predominant causes of liver disease in both group. There were no relevant differences between the two groups with respect to previous anti tumor therapy for HCC. Two (29%) patients had undergone surgery for HCC, and six (86%) had received prior transarterial chemoembolization therapy.

**Table 1:** Patients Characteristics.

	*Sorafenib Group*	*Control Group*
*Patients*	*7*	*9*
Age (years)	62.8 ± 15.7	52.9 ± 11.4
Sex (%) Male/Female	57/43	100/0
ECOG Performance status (%)		
0	4	7
1	3	2
≥2	0	0
BMI (kg/m^2^)		
≤18	1	1
18–25	3	5
>25	3	3
Child-Pugh classification		
A/B/C	5/2/0	5/4/0
Meld Classification<910–19>20	430	450
Cause of Cirrhosis		
- Hepatitis C only	3	3
- Hepatitis B only	2	0
- Alcohol only	1	3
- Other	2	3
Oesophageal Varices		
Grade 0/I/II	1/3/3	1/4/4
Advanced Hepatocellular Carcinoma	7	-
Prior therapy		
- Surgical resection	2	
- Locoregional therapy		
- Transarterial Chemoembolization	6	
- Radiofrequency ablation	1	
- Systemic anticancer therapy	1	

### Toxicity

All patients from sorafenib group reported cutaneous adverse events and one patient developed a grade 3 hand-foot reaction necessitating discontinuation of sorafenib. There were no reports of hypertension or renal toxicity. In both groups, no patients had deterioration of liver function, history of esophageal variceal bleeding or an introduction of a beta-blocker therapy during the period of monitoring. None of the patients were rehospitalized for reasons related to acute toxicity of sorafenib. Either in sorafenib group or in control group, no patient experienced specific cirrhosis complications such as acute oesophageal variceal bleeding or renal dysfunction during the study.

### Blood Flow

**Figure 2:**
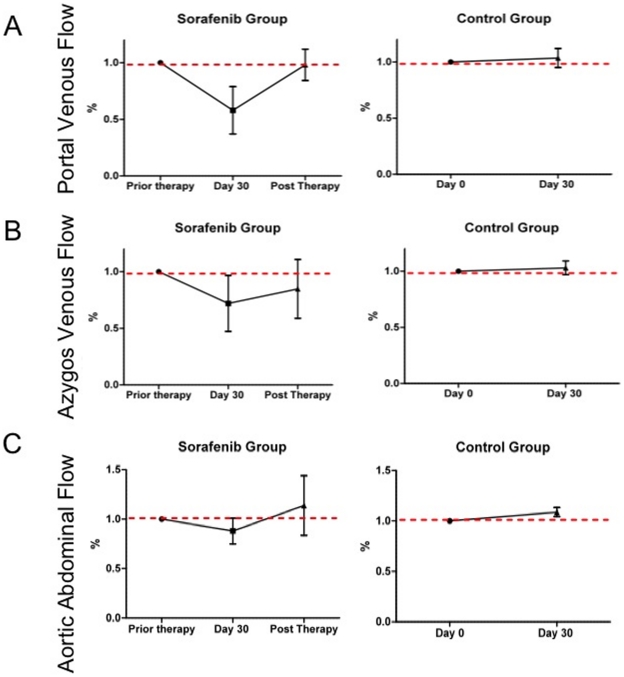
Modification of Blood flow measured on Magnetic Resonance Imaging under sorafenib therapy and after withdrawal. (a) Portal Venous flow, (b) Azygos Venous flow, and (c) Abdominal Aortic Flow.

At baseline, all patients had portal venous blood flow, values consistent with those observed in control group or previously described in cirrhotic patients (Table 2). [25] Patients from control group had no modification of porto-collateral circulations during the period of monitoring while patients under sorafenib therapy had a decrease in portal venous flow of at least 36% (Fig. 2a). At withdrawal, portal venous flow seeks to recover values before sorafenib. In contrast, no specific change was observed in the azygos vein or the abdominal aorta (Fig. 2b, c).

**Table 2:** Venous Flow at base line.

	*Sorafenib Group*	*Control Group*
*Magnetic resonance imaging*	*7*	*9*
**Portal Vein**		
Portal Flow (ml/s)	6.2±2.9	7.2±3.9
Portal Velocity (cm/s)	8.5±3.9	9.1±3.6
Peak Portal Velocity (cm/s)	11.7±4.7	13.8±3.9
**Azygos Vein**		
Azygos Flow (ml/s)	2.9±2.6	2.4±1.2
AzygosVelocity (cm/s)	13.6±4.7	10.4±3.4
Peak Azygos Velocity (cm/s)	25.8±6.3	20.4±7.0
**Abdominal Aorta**		
Aortic Flow (ml/s)	36.1±19	55.9±20.6
Aortic Velocity (cm/s)	13.4±4.2	23.5±6.2
Peak Aortic Velocity (cm/s)	53.5±19.8	70.6±16.4

Results are expressed as mean ± SD.

### Anti-Tumor Activity

At the time of analysis, two patients were still alive. At first assessment, patients achieved either a partial response or stable disease. Median progression free survival and overall survival were 153 days and 301 days respectively.

## Discussion

We report a significant reduction in portal venous flow (54% of mean portal venous flow) in seven patients with advanced HCC receiving sorafenib. Sorafenib is a potent multikinase inhibitor that targets the Raf/MEK/ERK pathway, as well as VEGFR1/2/3, PDGFR-β, KIT, Flt-3, and RET,[11] and has been approved in several countries worldwide for the treatment of renal cell carcinoma and HCC.[10], [13] In the SHARP study, sorafenib was both effective and safe in patients with advanced HCC,[10], [11], [12] but Child–Pugh liver function class B or C was an exclusion criterion.[10] Importantly, this classification does not evaluate the severity of portal hypertension, but reflects the extent of biological and clinical complications of cirrhosis. [26]

Receptor tyrosine kinase inhibitors have begun to receive a greater attention as a potential therapy in the treatment of portal hypertension and cirrhosis. [9] In our series, we highlight for the first time the positive side effect of sorafenib on portal hypertension. Our study confirms in the clinical setting the findings of previous preclinical studies. However, the generalization of our results is made difficult by the small population size. Therefore, a larger study is warranted to confirm our results. Our data suggest a beneficial effect in a one-month survey, but data are lacking on sorafenib long-term effects on portal venous flow. Also, one patient out of seven received a lower dose of sorafenib, showing a maintained effect on portal venous flow even at low doses. This case raises the question about the optimal dose to control portal venous flow, which could be explored in a phase I study.

In sorafenib group, 3 patients (43%) received 50% of the planned dose, resulting in a mean decrease of the portal venous flow of 32%. Those data suggest that a smaller dose of sorafenib than the one validated for HCC is able to decrease portal venous flow and modulate porto-collateral circulation.

Portal hypertension is the most important complication and the leading cause of mortality worldwide in patients with chronic liver diseases. [27] A characteristic feature of portal hypertension is the development of hyperdynamic splanchnic organs draining into the portal vein and subsequent portal venous inflow. Fernandez et al. [28], [29], [30] demonstrated that an increase in the splanchnic vascular bed size mediated by a VEGF-dependent angiogenic process contributed significantly to increased overall blood flow in splanchnic tissues in animal models of portal hypertension.[28], [29], [30] Interestingly, Mejias et al. showed that the effect of sorafenib daily were observed not only in the intrahepatic circulation, but also in the systemic and collaterals circulations suggesting the benefit of sorafenib in portal hypertension [8]. In our study, all patients receiving sorafenib therapy decrease portal venous flow and mean portal venous flow recovers its initial after sorafenib withdrawal. Those data are not related with a degradation of cirrhosis because no deterioration of the liver function was observed within the period.

Anti-VEGF agents are associated with an increased risk of bleeding. Most bleeding events are mild (grade 1–2), and may occur at any site (not specifically in the gastro-intestinal tract) [31]. On the other hand, cirrhotic patients do present a higher risk of bleeding events than the general population. As an illustration, in Abou-alfa study focusing on this frail population, only one out of 137 patients receiving sorafenib develops a grade 5 intracranial haemorrhage [32]. Also, patients receiving sunitinib do present a higher risk of bleeding events than those receiving sorafenib [31]. Je et al, in a systematic review and a meta-analysis of clinical trials showed for all grade bleeding a relative risk of 1.86 (1.33–2.6, p<0.0001) and for high grade bleeding a relative risk of 1.16 (0.57–2.35, p = 0.688) [31]. Indeed, bleeding events were not observed in our study, possibly due to the small size of patients' population.

VEGF-dependent angiogenesis plays a crucial role in the formation of portal collateral vessels.[28], [29], [30] Interestingly, Fernandez et al. suggested that the dual inhibition of VEGF- and PDGF-signaling pathways significantly reduced splanchnic neovascularization and pericyte coverage of neovessels, and translated into hemodynamic effects as a 40% decrease in portal pressure in a rat model.[33]

Meijas et al. demonstrated beneficial effects of sorafenib on intra-hepatic and portal circulations in cirrhotic rats.[8] It has also been shown to reduce portal pressure, superior mesenterial artery blood flow, and porto-systemic collateral blood flow in non cirrhotic rats with prehepatic portal hypertension, without affecting systemic hemodynamics.[18] Similarly, our present data suggest that sorafenib decreases portal venous blood flow without modifying aortic blood flow or azygos venous blood flow in patients with advanced HCC. Since HCC is a common complication of advanced cirrhosis, sorafenib is currently being used in patients with portal hypertension. Sorafenib should therefore be considered as an option to decrease portal hypertension in cirrhotic patients with advanced HCC.

Although beta blockers and endoscopic therapy are the only therapies proven to prevent portal hypertension induced bleeding, our data suggest that sorafenib might have a positive effect on portal venous flow. Hence, further studies should evaluate the impact of sorafenib compared to that of beta-blockers.

The dual inhibition of the VEGF- and PDGF-signaling pathways induced by sorafenib likely accounts for the decrease in portal venous flow observed in our patients. It has been suggested that the VEGF-signaling pathway is required not only for the development but also for the maintenance of portal hypertension.[8], [33] Consequently, inhibition of this pathway results in a significant attenuation of portal pressure and in reversal of the hyperdynamic splanchnic circulation in rats with advanced portal hypertension.[33] Those findings are consistent with our present data in humans, indicating up to an 84% decrease in mean portal venous flow within 30 days on sorafenib therapy, and a complete reversible effect in portal venous flow 30 days after sorafenib withdrawal.

Ebos et al. [34] have shown in preclinical models that priming with anti-VEGF agents promote tumour growth and dissemination. These findings suggest that short-term anti-VEGF therapy induces a “metastatic conditioning” in healthy organs. Hence, one could expect that short-term treatment of cirrhotic patients with sorafenib could promote tumour growth of a forthcoming cancer. So far, portal hypertension therapies such as beta blockers are introduced as soon as the portal hypertension is diagnosed, and pursued continuously until death. As well, in line with the results of Ebos et al. [34], we postulate that sorafenib might be administered indefinitely, thereby avoiding the deleterious effects observed at withdrawal of anti-VEGF therapy.

Sorafenib is the first drug to demonstrate a survival benefit and manageable side effects in patients with advanced HCC Child–Pugh class A. [10] Our data in patients with advanced HCC show a consistent decrease in portal pressure within 30 days of therapy, suggesting that sorafenib may also represent an option for patients with portal hypertension Child–Pugh class A and B. These findings deserve further investigation in larger prospective trials.
